# Revitalizing Brazil’s contribution to the International Society for Heart and Lung Transplantation Lung Transplant Database

**DOI:** 10.36416/1806-3756/e20240222

**Published:** 2024-11-16

**Authors:** Diego Corsetti Mondadori, Flavio Pola dos Reis, Fernando Antibas Atik, Luciana Bertocco de Paiva Haddad, Paulo Manuel Pêgo-Fernandes

**Affiliations:** 1. Grupo de Transplante de Pulmão, Serviço de Cirurgia Torácica, Hospital de Clínicas de Porto Alegre - HCPA - Porto Alegre (RS) Brasil.; 2. Grupo de Transplante de Pulmão, Disciplina de Cirurgia Torácica, Instituto do Coracao, Hospital das Clinicas HCFMUSP, Faculdade de Medicina, Universidade de Sao Paulo, Sao Paulo (SP) Brasil.; 3. Divisão de Transplantes, Serviço de Cirurgia Cardiovascular, Instituto de Cardiologia e Transplantes do Distrito Federal, Brasília (DF) Brasil.; 4. Divisão de Transplantes de Fígado e Órgãos Abdominais, Departamento de Gastroenterologia, Hospital das Clínicas, Faculdade de Medicina, Universidade de São Paulo - HCFMUSP - Sao Paulo (SP) Brasil.; 5. Associação Brasileira de Transplante de Órgãos - ABTO - Sao Paulo (SP) Brasil.; 6. Disciplina de Cirurgia Torácica, Instituto do Coracao, Hospital das Clinicas HCFMUSP, Faculdade de Medicina, Universidade de Sao Paulo, Sao Paulo (SP) Brasil.; 7. Departamento Científico, Associação Paulista de Medicina - APM - Sao Paulo (SP) Brasil.

## TO THE EDITOR:

The International Society for Heart and Lung Transplantation (ISHLT) is a professional, multidisciplinary, nonprofit organization dedicated to improving the care of patients with advanced heart or lung disease through transplantation, mechanical support, and innovative therapies by means of research and education. Currently, with more than 3,000 members across over 50 countries, the ISHLT mission is built on three pillars: education, research, and publications.^1^ The registration of transplant data is part of the institutional mission.

Since its inception in 1983, more than 220,000 lung, heart, and heart-lung transplants have been submitted to the ISHLT registry, involving approximately 260 lung transplant centers and 481 heart transplant centers across 47 countries. The goal of the ISHLT Registry is to improve the care of patients with terminal lung or heart diseases.^2^ It has succeeded in recruiting members from various regions and has catalyzed clinical and scientific improvements and collaborations. However, challenges in data reporting have been observed in Brazil. Initially, the responsibility for providing data was of the participating centers, which, due to the voluntary nature of participation, resulted in heterogeneous commitment. The lack of human resources for data reporting, prevalent in most hospitals in the country, significantly impacted the way data were reported. Additionally, there were no penalties for failing to send data, causing some centers to cease reporting over time.

In 2021, the ISHLT Registry was halted for regulations concerning data sharing and improvements in evaluated standards. Both data fields and the upload process were updated. In 2024, information will be shared only between the ISHLT Registry and national and regional registries, no longer between hospitals or institutions.

Considering that Brazil is the country with the highest number of organ transplants in Latin America, it is natural that our transplant society-the *Associação Brasileira de Transplante de Órgãos* (ABTO, Brazilian Association of Organ Transplantation)-has a transplant registry. This registry is the *Registro Brasileiro de Transplantes* (RBT, Brazilian Transplant Registry). Established since 1996, it is the official registry of all transplants performed in the country, including lungs, kidneys, liver, heart, pancreas, bone marrow, cornea, bones, and muscle tissues.^3^ Therefore, to maintain Brazil’s participation in the new ISHLT registry format, it is reasonable that the ABTO be the national association of choice for representation.

**Figure 1:**
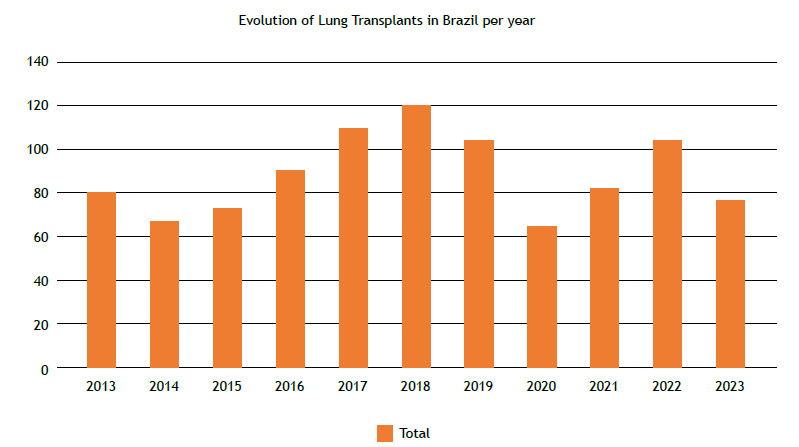
Evolution of lung transplants in Brazil per year.

The RBT also includes sections on pediatric transplants, waiting list times, and organ donations. The publication is made quarterly with partial data, and annually with complete data. These publications are crucial for decision making in organ transplant programs in the country. Lung transplant centers participate voluntarily, contributing with mandatory variables (date of birth, gender, ethnicity, ABO group, underlying diseases, and current status) and optional variables (serology, need for pre-transplant cardiopulmonary support, need for ventilatory support, single or bilateral transplant, retransplantation, and donor conditions such as days of orotracheal intubation, cold ischemia time, Pao_2_/Fio_2_ ratio, donor serology, and prioritization). In 2023, 78 lung transplants were performed in three states (Rio Grande do Sul, Rio de Janeiro, and São Paulo) in Brazil, corresponding to 0.4 transplants per million population. This number is lower than the 106 performed in 2022, the year post-pandemic recovery from SARS-CoV-2, and also lower than the 120 transplants performed in 2018. Of the 78 transplants, 15 and 63 were unilateral and bilateral, respectively. One-year, three-year, five-year, and ten-year survival rates were 70%, 54%, 44%, and 22% for unilateral transplants and 68%, 58%, 52%, and 38% for bilateral transplants.^4^ Figure 1 shows the evolution of lung transplants in Brazil by year until 2023.

It is evident that we must always strive to improve the national lung transplant database. Such an initiative will make information about our reality clearer and more significant, promoting higher quality programs and new discussions about funding transplant centers in public and private health care systems. The leaderships of the ABTO and the ISHLT have already signed a data-sharing agreement. Thus, transplant centers will continue to fill in their own data online in the RBT in Portuguese. Once a year, the ABTO will transfer all data, translated into English, to the ISHLT Registry, securely and anonymously, with no patient or hospital identification, within the strictness of data protection laws. This ensures the opportunity to participate in the global database actively so that we will have international outcome standards to compare with ours. Finally, it opens the opportunity for education and research, focusing on national needs.

To make this possible, the RBT has been upgraded to version 2.0. This is particularly important to comply with legal data protection standardizations, confidentiality, privacy, and information integrity. During the modernization of our registry, new variables were included with the same codifications as the ISHLT Registry, which will facilitate the data export process. The progressive incorporation of new variables is the plan for the future.^5^

To achieve success in this project, it is essential that all centers proactively engage, aiming to contribute and improve the national and international registries. The benefits resulting from this collaboration will bring advantages for both our patients and all of us.
